# Dental Hints Department

**Published:** 1906-03

**Authors:** B. H. Teague

**Affiliations:** Aiken, S. C.


### DENTAL HINTS DEPARTMENT.

Conducted by B. H. Teague, D. D. S., Aiken, S. C., to whom all contributions to this department should be sent.

It is a dangerous practice to operate at the chair and run a vulcanizer at the same time.

How many of our readers are studying subjects for their papers for the society meetings soon to be held?

The National Dental Association meets in Atlanta, Ga., this year, September.

Neuralgia.—A hot water bottle covers a multitude of indications; a hot mustard footbath is singularly effective.—*Alk. Clinic.*

To what has the grand official science reduced itself—docketing patients and maladies and cataloguing new medicaments?—*La Dos.*

One part kerosene, one soft soap with six of water forms an effective preventive of mosquito and gnat attacks.—*Hammond, Alk. Clinic.*

F. T. Lord has proved that flies carry and deposit tubercle bacilli in a virulent degree of development. Darn a fly anyhow.—*Alk. Clinic.*

The liver arrests poisons; we must stimulate its action by proper therapeutic agents; also skin, lungs, bowels, kidneys.—*Field, Alk. Clinic.*

Strychnine, camphor and caffeine are the most valuable cardiac stimulants that we have; latter aids tissue formation.—*W. H. Porter, Alk. Clinic.*

Before filling with gold thoroughly cleanse tooth and adjacent tooth with alcohol. It may save you some black marks in the good book.—*Dr. L. A. Badger, Titusville, Pa.*

Dr. Sibley added that the same object could be attained (according to the late Dr. Palmer, he thought) by treating the freshly exposed surface with silver nitrate.—*Of. and Lab.*

SHOEBLACKING.—Dr. Beebee had found common shoeblacking a perfect preventive of adhesion of fusible-metal to gold-shells when swaged over forms of that material.—*Of. & Lab.*

IMPROVED SOLDERING OR TINNING ACID.—Into 1 lb. muriatic acid put all the zinc it will dissolve and 1 oz. sal. ammoniac. Add as much clear water as there is of the acid.—*Pop' Mech's.*

COLD SOLDERING.—Dr. Beebee also said that fresh amalgam could be made to stick to an old amalgam filling by merely coating the freshly exposed surface of the latter with hydrochloric acid.

To distinguish steel from iron apply a drop of nitric acid and let it remain for a moment, then rinse with water. If the metal is iron, a whitish-grey spot will remain; if steel a black stain.—*Popular Mechanics.*

SENSITIVE DENTIN.—Jarring the tooth with an automatic mallet, having a blunt-point planer in the cavity aids materially in inducing the penetration of fluids into the dentin.—*N. C. Leonard, Dental Headlight.*

TREATMENT OF VERY SORE TEETH.—I have had great help from the use of a string tied around the tooth, instructing the patient to draw on it until it is real tight. The effect will be a revelation.—*F. Milton Smith, International Dental Journal.*

LAC EDGE OF INLAY.—In inlay work, paint the peripheral edges of the matrix with shellac before placing the body. The shellac will burn out and prevent warping of the inlay. The crevice is filled at the second placing of body.—*Western D. Jour.*

I do not believe that any suspicious looking cracks will appear in enamel after polishing a gold filling if the cavity has been properly prepared and the gold matted well against the margins before final condensation.—*Dr. L. A. Badger, Titusville, Pa.*

For tightening screw connections, dissolve powdered shellac in 10 per cent ammonia and paint the mass over the screw threads after they have been thoroughly cleaned; then screw the fitting home. The joint will be impervious to hot or cold water.—*Popular Mechanics.*

Pulp Extirpation.—Where immediate extirpation is intended, if a pellet of cotton is placed over the rubber in the cavity a much better pressure can be obtained, as the cotton prevents the rubber from spreading so much under the instrument.—*W. A. Brownlee, Dental Brief.*

Watchmakers' Oil.—In a bottle about half full of good olive oil, put thin strips of sheet lead. Expose to the sun for a month's time. Then pour off the clear oil. This is a cheap method of making a first-class oil for any light machinery. The oil will not corrode or thicken.—*Popular Mechanics.*

For Rough Hands.—Take a four-ounce bottle and put in same three ounces of glycerine, one ounce alcohol, and from twenty to thirty drops of carbolic acid. After washing the hands and while they are a little damp apply a few drops and thoroughly rub it in. A good time to use it is at night.—*Pop' Mech's.*

Tooth Massage.—It is to my mind a question whether it is advisable by excessive use of pumice to rub away the enamel cuticle at points of the tooth which are susceptible to decay as I have found that the destruction of this membrane involves an increase of susceptibility of the tooth to decay.—*W. D. Miller, British Dental Journal.*

If possible a mouth should not be rendered edentulous, but in each jaw two roots should be left and utilized to support the plate. A satisfactory method is to fit each root so left with a gold cap and tube, into which fit a pin attached to the plate. The stability which even one root so treated will give to an entire denture is surprising.—*Dental Record.*

Vulcanizer Explodes.—The explosion of a vulcanizer in the office of Dr. W. R. Clark, at Youngstown, Ohio, almost resulted in death. The top blew up, striking the ceiling above and throwing James A. Campbell, president of the Youngstown Sheet and Tube company, from the chair, so great was the force of the impact.—*Amer. Dental Jour.*

Amalgam Carrier.—As an amalgam carrier he had made good use of a double-end file of a pattern that filled an important place in the operating outfits of long ago. The pod-like ends, somewhat larger than wheat grains, he coated with soft solder. As a picker up and conveyor of pieces of amalgam it had few equals and no superiors.—*Of. & Lab.*

Pain After Tooth Extraction.—The extraction of an abscessed tooth is generally followed by great pain. I have found lysol to be the ideal remedy in such conditions, placing it undiluted in the socket. It will relieve the pain immediately, help to check the hemorrhage and establish antiseptic conditions in the socket.—*G. B. Winter, Dental Era.*

A Good Obtundant.—I find that a saturated solution of potassium carbonate in glycerine, as given in the September number of *The Dental Summary,* very effective for sensitive dentine. I apply to cavity on cotton and let remain from ten to fifteen minutes. It can be used freely in the open mouth, as it is non-poisonous.—*Dr. L. W. Jordan, Summary.*

Pulp Removal.—The important point to remember in opening up a cavity in a tooth containing a dead pulp, is the danger of forcing infected pulp detritus through the end of the root; consequently it is important to bathe the interior of the pulp-chamber with a 10 per cent solution of formaldehyde before instrumentation is begun.—*M. L. Rhein, Dental Digest.*

Gold Filling in the Appendix.—A patient of mine, Mr. J. A. Halfast, reported to me that an operation for appendi citis on his brother last summer disclosed the fact that the etiology lay in a large gold filling which had lodged in the appendix and a sharp edge had perforated the appendix wall. The patient did not recover.—*Dr. L. A. Badger, Titusville, Pa.*

To obliterate the disagreeable odor which is taken up by rubber and wooden handles when using formaldehyde sterlizers, use a solution of 5.20 per cent camphenol and immerse the instruments. The water takes up the formaldehyde to a certain degree and the camphenol leaves a very faint odor which is not disagreeable to the patient.—*Dr. L. A. Badger, Titusville, Pa.*

Oral Hygiene.—As an especially effective wash to decrease the ravages of caries, mercuric chlorid in the strength of one to twenty-five hundred forms a valuable constituent of the prophylactic dentist's armamentarium. In many mouths it will not be indicated, but in those mouths passing through the stage of extreme susceptibility it will be prescribed.—*Geo. E. Hunt, Dental Digest.*

Care of Tooth Brush.—The tooth brush may be kept sterile by taking a large size test tube and constructing it about one inch from the bottom, providing it with a rubber cork as a stopper and placing a little formalin tablet or formaldehyde in the lower chamber and the brush in the upper chamber. It will then become sterile without injury to the brush.—*Dr. T. H. Hardgrove, Review.*

Massaging the gums with sulpher three or four times a day has been advised by Dr. Harlan, or a paste may be made from the following formula: Boric acid, 2 oz.; tanic acid, 1 oz.; glycerine, 1 oz.; and tincture of cantharides, 20 gr., the latter being preferable where there are many amalgam fillings present, as the sulpher may have a discoloring effect upon the amalgam.—*Dr. T. H. Hardgrove,* ***Review.***

Dyspepsia.—Fermentative dyspepsia with acidity of the stomach and heartburn is a pathological condition most readily relieved by the administration of twenty or thirty grains of pancreobismuth with pepsin. This, mixed with one-third of a glass of water after meals will seldom fail in giving temporary relief, and if continued for some time, permanent relief will be had.—*F. H. Benedict, M. D., Med. Brief.*

Amalgam—Cut and Color.—Other things being equal, coarsely cut alloy discolors in less time and more deeply than the same alloy when cut fine; but the difference may be reduced, even obliterated, if the coarsely cut product is subjected to prolonged treatment in the amalgamating process. This is easily demonstrated in alloys that become smooth and pasty only when long kneaded in the palm of the hand.—*Of. & L.*

Preventive Treatment: Nitrate of Silver.—It is safe to say that the nitrate of silver leads to the formation of secondary dentin in so far as it converts the acute with the chronic form of decay. The medicament consequently has a double action, in that it renders the decalcified dentin more or less impentrable to acids, and also facilitates the interposition of a layer of secondary dentin as the part of the living pulp.—*W. D. Miller, British Dental Journal.*

Outline of Plate.—All plates should be worn as high as possible all around, but always higher over the cuspid teeth than elsewhere. To know where to locate the high points, use a pair of compasses and measure the teeth which you are to use, from the median line to center of cuspid, transfer the measurement to the die and from that to the plate and trim accordingly, dropping suddenly back of the high point to give free play to the muscles.—*Dr. L. P. Haskell, Dem. Magazine.*

Cold Abscess of the Tongue.—An eight-year-old girl presented a small abscess the size of a pea, on the middle of the dorsal aspect of the tongue, near the median line. It had developed slowly and painlessly, there was no suspicion of tuberculous affection. The pus taken from the abscess had the gross appearance of tuberculous matter, but the microscope revealed no micro-organisms. The rare occurrence of such cases makes this report interesting.—*Dr. Mercade, Gazette des Hopitaux.*

Anti-flux.—The speaker said further that he had found it extremely difficult, following the prescribed methods of applying anti-flux, to keep borax from binding backings to porcelain faces. He now swaged the backings, which he always made of two thicknesses of plate, to the porcelains, removed them, painted the to-be-applied surfaces with a thin mixture of chalk and water, replaced the backings and soldered, with the result that there was no adhesion of gold to the porcelain, therefore no fracture of the facing.—*Of. & Lab.*

Anti-mourning Method.—Dr. Belcher again called attention to the lining with heavy gold foil of cavities to be filled with amalgam. If properly done much if not all of the staining of enamel, or showing through where this tissue was thin, could be prevented. To make the gold adhere to the tooth he employed an adhesive, such as copal disolved in ether, and to prevent the amalgamation of the gold he gave that a coat of the adhesive also. Balsam in chloroform answered as an adhesive, but copal in ether had the advantage of quickly drying.—*Of. & Lab.*

To Purify the Office.—The following antiseptic combination is recommended as a vapor in the office or sickroom:

℞ Eucalyptol, ʒ iiss.

Ol. thymi,

Ol. limonis,

Ol. lavendulæ flor., aa ʒ j.

Alcoholis, ℥ iv.

M. Sig. One teaspoonful to be floated on a pint of boiling water and allowed to evaporate.—*Jour. A. M.*

"Sure Toothache Cure."—The Practical Druggist gives the following as a "sure toothache cure":

Creosote
Chloroform
Oil cloves
Oil peppermint, of each ....................5 drams
Oil camphor (essential)
Carbolic acid, in crystals, of each ..........6 drams

It really looks as if it would do it, if anything would.

Toothache.—The following will be found very useful to physicians who are called upon to treat toothache.
blister the mucous membrane of the mouth and rar[illegible]
to stop the pain:

℞—Phenol ..............................1 drachm.
Ol. Gaultheriæ ........................2 drachms.
Ol. Cinnamoni .........................3 drachms.

M. Sig.: Saturate a pledget of cotton and insert in the well cleansed cavity of the tooth.—*Geo. R. Pennington, M. D., Med. Brief.*

Articulation.—There are more failures from faulty articulation than from any other cause. Never allow the six anterior teeth to come in contact. Be sure the two sides occlude exactly. If a second or third molar is tilted forward so as to be at an angle do not allow the upper teeth to meet it, as sooner or later the plate will be crowded forward. The pressure should be on the bicuspids and first molars. * * *

In arranging lower teeth in a full set, begin with the second bicuspids so as to secure correct closure of masticating surfaces. Then if necessary, select narrower fronts as they are often too wide for the uppers.—*Dr. L. P. Haskell, Dental Magazine.*

Antiseptic Mouth Wash.—The following is an excellent formula according to *Merck's Report:*

℞ Formalin, *m* v.

Tinct. benzoin, fl. ℥ iij.

Tinct. myrrh, fl. ʒ j.

Ol. of peppermint, *m* iij.

Ol. of anise, *m* ij.

Ol. of cassia, *m* j.

Ol. of cinnamon, *m* xv.

Alcohol, fl. ℥ ij.

M. Sig. Use as a mouth-wash once daily.—*Ex.*

Piles.—It may not be known to the readers of the Medical Brief that the most distressingly painful hemorrhoids may be cured by the internal use of the root of the beautiful peony. It cures protruding bleeding piles when they are extremely sensitive to touch, and when the pain comes after stool. If there is fissure or ulceration, this remedy is all the more promising. If there is much induration of the edges of the fissure, the relief will come from the persistent use of this excellent remedy. It has cured even in decoction. The dose should be exceedingly small.—*James Tyler Kent, Med Brief.*

Relative to the payment for our supplies, while there are some practitioners who are prompt, there is too much carelessness shown by many in this particular. Comparatively few take advantage of the ten per cent discount offered by the dental depots in paying for one hundred dollars' worth of supplies in advance. This represents quite a saving, and it would be a very difficult matter for any of us to invest our money to better advantage. It is a very desirable thing to do, and with responsible houses it is perfectly safe. When favors are shown in the courtesies of commercial relationship the dentist who discounts his bills is quite certain to receive such attention.—*Dr. G. F. Burke, Register.*

Eyestrain.—"The common symptoms of eyestrain are, according to Dr. Gould, headache, insomnia, biliousness, nervousness, dejection, 'indescribable suffering,' cessation of all symptoms if the eyes are rested, and final spontaneous cure at about the age of 60, when the paralysis of accommodation occurs. He states that an abnormally large proportion of criminals have serious defects of the eyes, and 98 per cent of the epileptics he examined suffered from astigmatism. Brain fag and migrain or sick headache are nothing more than eye-strain, and may be absolutely cured by glasses, and even temporarily relieved by a few drops of belladonna, which dilates the pupil and paralyzes accommodation."—*Of. & Lab.*

LYMPHANGITIS.—Lymphangitis is a usual accompaniment of alveolar abscess, and is often quite pronounced in the lighter forms of the affection. This often appears early in its progress, and is as often lost sight of in its after progress, though still an important complication. It is the direct result of the poisoning of the lymph streams by the products of inflammation or by-products of pus formation, or, in either case, by microbic poisons. It is seen most in swelling and soreness of the lymph glands just under the lower maxillary bone, to the rear of the submaxillary gland and in the floor of the mouth; later, anywhere about the angles of the neck. In persons of ordinary health these glands, though they become very sore and often distressing, rarely suppurate. If, however, the glands are already tuberculous, suppuration may occur as a sequel and give trouble.—*Dr. G. V. Black, Ex. Northwestern.*

SENG.—The existing prejudice against Ginseng is due to the fallacious claims made for it by the Chinese. However, if one will judge by false claims only and never see the good in a drug, he will find his materia medica greatly curtailed. When the practical application of a drug demonstrates its usefulness, not in one case, but in many cases, as Seng has done for me, I am ready to subscribe to its therapeutic value, notwithstanding prejudice. Theory or prejudice have never yet cured a patient. In my experience Seng will stimulate all secretory glands and after cleansing with necessary eliminants, it will render the alimentary tract physiological. In the treatment of digestive derangements, whether primary or symptomatic, I find that in connection with a rational diet and indicated correctives, Seng will stimulate gastric secretion. In anemia and chlorosis, where the motor functions of the stomach are not disturbed, yet there is a decrease in secretion of gastric juices, it is of greatest value as collateral treatment.—*J. J. Hoffman, M. D., Clinique.*

